# The Random RFPA Method for Modelling Rock Failure

**DOI:** 10.1007/s00603-025-04400-3

**Published:** 2025-01-27

**Authors:** Bin Gong, Tao Zhao, Indrasenan Thusyanthan, Chun’an Tang, Gordon G. D. Zhou

**Affiliations:** 1 Department of Civil and Environmental Engineering, Brunel University of London, London, UB8 3PH UK; 2 State Key Laboratory of Geomechanics and Geotechnical Engineering Safety, Institute of Rock and Soil Mechanics, Chinese Academy of Sciences, Wuhan, 430071 China; 3 Gavin & Doherty Geosolutions, London, W1W 7LT UK; 4 State Key Laboratory of Coastal and Offshore Engineering, Dalian University of Technology, Dalian, 116024 China; 5 Key Laboratory of Mountain Hazards and Earth Surface Process, Institute of Mountain Hazards and Environment, Chinese Academy of Sciences, Chengdu, 610000 China

**Keywords:** Rock failure, Material heterogeneity, Random field theory, Crack propagation, Acoustic emission

## Abstract

The random rock failure process analysis (RRFPA) method was developed in this research to characterize the material spatial variability and uncertainty in rock failure modelling. The random field theory (RFT) was integrated with the traditional rock failure process analysis (RFPA) to model rock heterogeneity. In this approach, the variation of rock properties is represented as a function of relative distance, such that the influence of material intrinsic correlation on its fracturing behaviour can be appropriately captured. To validate the theory, 300 RRFPA simulations were conducted to investigate the failure characteristics of rock samples under compressive loading. The results showed that by incorporating a spectrum of material properties, the numerical outcomes exhibited distinct upper and lower bounds of stress across all testing scenarios, closely aligning with the experimental relationships. The histograms for uniaxial compressive strength and elastic modulus showed that both properties followed normal distributions, with the average values of 10.099 MPa and 1.818 GPa, respectively. The corresponding coefficients of variation were 0.450 and 0.038. The localized failure tended to result in a more rapid release of acoustic emission energy, but generated smaller cumulative energy compared to the overall failure pattern. In general, the maximum relative error of the RRFPA model was only 0.66% for uniaxial compressive strength, elastic modulus, and critical axial strain.

## Highlights

- The random rock failure process analysis method was developed by coupling the rock failure process analysis method and random field theory.
- The RRFPA method can characterize the spatial material variability and uncertainties in rock failure modelling.
- The influence of material intrinsic correlation on the fracturing behaviour and failure modes can be appropriately captured.
- In the RRFPA analyses, the results exhibited distinct upper and lower bounds of stress distribution across all testing scenarios.

## Introduction

Rock failure poses significant risks to both infrastructure and human life through various mechanisms, often leading to catastrophic damages (Bai et al. 2024; Ma et al. 2024; Zhao et al. 2024). Early detection and prevention of potential rock failures are crucial in reducing the maintenance or reconstruction costs for infrastructures such as buildings, bridges, tunnels, dams, and highways. Relevant studies are required to assess the causes of rock failure, and to predict and mitigate natural disasters such as landslides, rockfalls, and debris flows (Tanyaş & Lombardo 2019; Zhao and Feng 2023). Additionally, human activities such as mining, blasting, and tunnelling can cause hazardous rock failures (Chen et al. 2023a; Liu et al. 2024; Wang et al. 2024; Yao et al. 2022). These activities can alter the natural geometry of rock formations and weaken the stability of surrounding rocks, resulting in rock fractures and collapses. The reduced rock strength, altered rock geometry, degraded rock quality and loading rate are key factors contributing to the instability of rock structures (Fakhimi et al. 2018; Zhang et al. 2021). However, comprehensively investigating the cross-scale mechanisms behind rock failure and understanding its impact on rock instability, as well as the associated energy losses of rock fragments, still remains a significant challenge.

From the perspective of rock mechanics, the physical and mechanical characteristics of rock exhibit substantial variations across the whole rock mass, owing to the diverse material properties of constituents and the complex formation history (Blair and Cook 1998; Ebner et al. 2009; Pinheiro et al. 2016). The intricate spatial microstructure of rock plays a pivotal role in determining its geotechnical properties, introducing factors such as discontinuity, inhomogeneity, anisotropy, and nonlinear elasticity. For instance, under external loading, rock damage often initiates at relatively weak locations due to the localized stress concentrations surpassing material strength (Karatela and Taheri 2018; Sun et al. 2024). Despite the widespread prevalence of spatial heterogeneity in rock properties, only a limited number of studies have addressed this phenomenon. In numerical analyses, rock properties can be effectively represented as multidimensional and multivariate random fields. The utilization of random field concept facilitates a consistent characterization of non-uniform material property. In this regard, the random field theory (RFT) has been widely used to characterize the spatial variability material properties in geosciences and geotechnical engineering (Casagrande et al. 2018; Fenton and Griffiths 2008; Liu et al. 2014, 2019). RFT is particularly valuable for modelling the variability in rock properties such as strength, permeability, and density, which are crucial for understanding and predicting the behaviour of geomaterials (Feng et al. 2023; Hu and Wang 2020). The theory employs statistical methods to generate random fields that reproduce the observed variability and spatial correlation structures. In the literature, various techniques such as Gaussian random fields, Gamma random fields and Markov random fields have been successfully applied to capture this variability (Fang & Liu 2022; Yang et al. 2023). By accounting for the spatial variability, random field models provide more realistic predictions of rock behaviour and geotechnical response compared to deterministic approaches. Furthermore, random field models allow the assessment of uncertainties associated with geological variability, providing engineers with the ability to quantify risks and make informed decisions in design and planning processes.

During the last few decades, the numerical analyses have significantly advanced the understanding of rock failure in various engineering and geological applications (Chen et al. 2022; Li et al. 2021; Ning et al. 2023; Qin et al. 2024). These analyses provide access to detailed insights into the internal response of rocks, including stress distributions, crack propagation paths, and fracture parameters, which are often difficult or impossible to be measured experimentally. Meanwhile, numerical models also facilitate sensitivity analysis by allowing researchers to systematically vary input parameters and observe their effects on fracture behaviour. This process helps identify critical parameters and uncertainties in the modelling process. Particularly, the rock failure process analysis (RFPA) method can account for rock heterogeneity in modelling the initiation, propagation, and coalescence of cracks (Feng et al. 2022; Gao et al. 2023; Wang et al. 2023a). The RFPA code has been applied to simulate the progressive failure of rock masses by many researchers (Chen et al. 2023b; Gong et al. 2022; Wang et al. 2023b; Yu et al. 2022). In this approach, the rock heterogeneity is considered by assuming that certain mechanical properties, such as Young’s modulus and strength of the elements within a model, conform to a Weibull distribution (Weibull 1951). However, while the statistical Weibull distribution can describe the non-uniform distribution of material properties to some extent (Gong et al. 2024), it could not fully capture the intrinsic correlating between these properties.

In this study, the random RFPA method (RRFPA) was developed by combining RFPA and RFT to provide an integrated approach for characterizing and quantifying the spatial material variability and uncertainties in rock failure analysis. Furthermore, it was utilized to analyse the mechanical responses of rock during the fundamental uniaxial compression tests, including the stress–strain relationships, fracture characteristics, acoustic emissions, and failure patterns. By adopting a repetitive simulation scheme in the random RFPA, 300 simulations were conducted with randomly distributed but spatially correlated material properties. This approach facilitated a statistical examination of the mechanical behaviour of rock, enhancing our understanding of the probability of rock failures.

## Methodology

### Random Fields of Material Properties

In this study, material properties were evaluated as spatial variables using the discrete random field approach. Within this framework, all material points were assumed to exhibit mutual correlations over a predefined length, which defined the scale of fluctuation. Both the elastic modulus and uniaxial compressive strength (UCS) were modelled as spatially non-uniform distributions in the analysis. To generate a standard random field realization, the covariance matrix was first constructed according to the autocorrelation function. Then, the Cholesky decomposition of the covariance matrix was performed, and the standard normal random matrix was multiplied by the Cholesky lower triangular matrix to form the standard Gaussian random field. After that, it was transformed into the non-Gaussian random field through the equal probability approach, i.e., the desired random field realization was obtained (Haldar and Babu 2008; Srivastava et al. 2010; Suchomel and Mašín, 2010). In this study, the exponential cosine autocorrelation function has been employed, as follows:

1 $$\rho \left({\tau }_{x},{\tau }_{y}\right)={e}^{\left(-\left(\frac{{\tau }_{x}}{{\delta }_{x}}+\frac{{\tau }_{y}}{{\delta }_{y}}\right)\right)}\times \text{cos}\left(\frac{{\tau }_{x}}{{\delta }_{x}}\right)\times \text{cos}\left(\frac{{\tau }_{y}}{{\delta }_{y}}\right)$$

where $\rho \left({\tau }_{x},{\tau }_{y}\right)$ represents the autocorrelation coefficient of the random field eigenvalues at two points *P*_*i*_ and *P*_*j*_ in the two-dimensional (2D) space; ${\tau }_{x}$ and ${\tau }_{y}$ represent the relative distances along the horizontal (*x*) and vertical (*y*) directions between *P*_*i*_ and *P*_*j*_, respectively; ${\delta }_{x}$ and ${\delta }_{y}$ represent the horizontal and vertical correlation lengths, respectively, characterizing the spatial autocorrelation degree of material properties. Clearly, the larger fluctuation range corresponds to the stronger spatial autocorrelation of material properties.

**Fig. 1:**
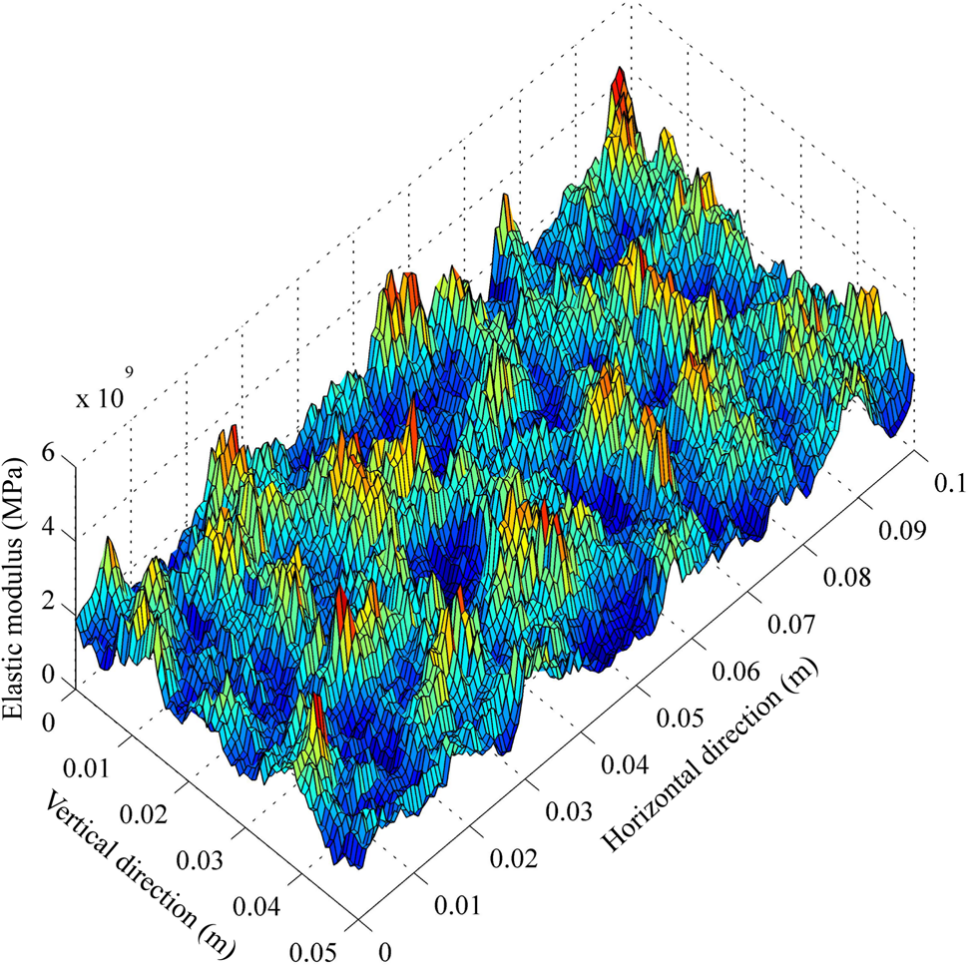
A typical realization of random field for elastic modulus fluctuation

Furthermore, the statistical mean value and coefficient of variation (COV) are two key parameters controlling the generation of random fields. COV, defined as the ratio of standard deviation to mean, stands as a pivotal metric for assessing variability. Then, according to the correlation between the coordinates of discretized elements and the generated random field realizations, the centre of each element was assigned a material coefficient (*α*). Consequently, precise material properties were deduced by multiplying this coefficient with the mean material properties, which underwent fine-tuning via iterative adjustments. These adjustments were conducted by aligning the outcomes from the numerical RRFPA simulations with the experimental data. During this iterative process, the specific parameters, such as the elastic modulus and UCS of the rock in all mesh elements, were adjusted to match the predetermined mean values. At the same time, consistency was maintained across multiple tests for other statistical parameters, including the COV and correlation length. Figure 1 shows a typical realization of random field with clear fluctuation of elastic modulus across the whole 2D section of the rock sample.

**Fig. 2:**
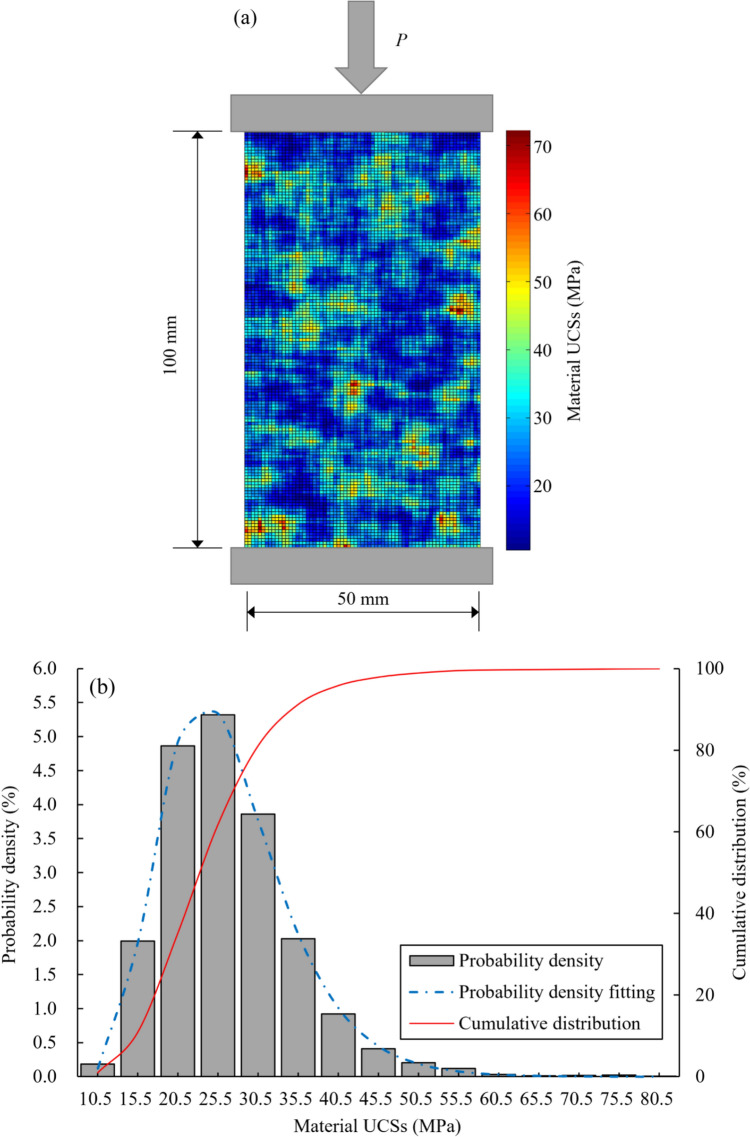
**a** Model configuration for 2D uniaxial compression test (note: the colour of mesh element represents the magnitude of material UCS); **b** probability distribution of material UCSs

To evaluate the performance of the proposed RRFPA method, the uniaxial compression tests conducted by Liu et al. (2015) were simulated. The 2D numerical model with the size of 50 mm × 100 mm was generated as illustrated in Fig. 2a, and the vertical load was applied at the top of the rock sample by controlling its axial downward displacement at a constant rate of 0.0001 m per iteration step. Simultaneously, the bottom was fixed along both horizontal and vertical directions. The prescribed statistical parameters of input parameters were selected based on Zhao and Liu (2020), as listed in Table 1. The probabilistic distribution of material UCSs of the rock sample is displayed in Fig. 2b. In this study, uniaxial compression tests were conducted under a repetitive simulation scheme, using the same model configuration but varying the random field distributions of material properties in each run. A total of 300 simulations were performed, enabling a comprehensive statistical analysis of rock behaviour. This approach effectively captures the inherent variability in the random fields across different simulations.

**Table 1:** The statistical parameters for input variables

Variable	Mean	Coefficient of variation	Correlation length
Elastic modulus	1.89 GPa	0.3	5 mm
Uniaxial compressive strength	27.76 MPa	0.3	5 mm

The proposed RRFPA method presents a significant improvement over the traditional RFPA approach, which typically relies on a single set of constant mesoscopic material properties. By incorporating the spatial variability of material properties across a range of random fields, the RRFPA method ensures that the mean material properties remain consistent throughout the simulations. This approach enables more accurate modelling of rock failure behaviour, accounting for the inherent variability in material properties within rock samples. Through the extensive statistical analysis, these simulations could possibly replicate the fundamental mechanical response of rock masses with comparable internal weak zones. Consequently, this research offers a more robust and reliable framework for characterizing the uncertainties and complex interactions inherent in specific rock materials.

### Failure Criteria

In the RRFPA method, each element is initially loaded in the elastic state, defined by its mechanical properties, including elastic modulus and Poisson’s ratio. Throughout the simulation, each element follows a linear elastic constitutive relation until it reaches a predefined damage threshold, at which point the onset of the softening phase begins. Extensive research has revealed the inadequacy of solely considering tensile crack opening or crack sliding to fully capture the intricate fracturing process (Hoxha and Homand 2000). Among the different crack evolution mechanisms, tensile opening and shearing stand out as predominant factors (Meglis et al. 1995). Therefore, the Mohr–Coulomb criterion with a tensile cut-off is chosen to analyse the primary modes of rock damage and fracture. The stress states of model elements should not exceed the spatial failure envelope described by the strength criterion. Within the damage zone forming at the current time step, the mesh element stress states should be on the failure envelope in the stress space. However, the yield strength of an element can be exceeded during the numerical modelling. Although such specific stress state cannot occur in practical scenarios, it emerges during the numerical calculations because of the constraints imposed by time step.

When subjected to uniaxial tension, the elastic-brittle damage constitutive relation at the mesh element level is employed, and the tensile damage function is defined as follows:

2 $${\sigma }_{3}\le {f}_{\text{t}}$$

where *σ*_*3*_ represents the minimum principal stress, and *f*_t_ denotes the uniaxial tensile strength. It is important to note that negative values are assigned to both tensile stress and strain.

Simultaneously, the Mohr–Coulomb criterion is utilized to determine if some elements are damaged under the applied shear loading stress, and the corresponding judgement can be carried out according to the following relationship:

3 $${\sigma }_{1}-\frac{1+\text{sin}\varphi }{1-\text{sin}\varphi }{\sigma }_{3}-{f}_{\text{c}}\ge 0$$

where *σ*_1_ represents the maximum principal stress; *f*_c_ and *φ* represent the UCS and internal friction angle, respectively.

### Damage Behaviour of Elements

According to the elastic damage mechanics, the elastic modulus gradually degrades as rock damage progresses, once the specific strength criterion is satisfied. The modified elastic modulus after damage is determined as:

4 $${E}_{\text{w}}=(1-w){E}_{0}$$

where *w* denotes the damage variable; *E*_w_ represents the modified elastic modulus after element damage occurs; *E*_0_ signifies the initial elastic modulus before element damage occurs.

At the initial loading stage, the constitutive relationship is linear-elastic with no damage under the uniaxial tension or compression stress. However, at a later stage, the constitutive equation is attributed to a specific residual strength after the failure criterion is satisfied. Furthermore, tensile damage will arise when the maximum tensile strain criterion is reached, and the damage variable *w* could be calculated using the following equation:

5 $$w = \left\{ {\begin{array}{*{20}c} 0 & {\varepsilon> \varepsilon_{{{\text{t0}}}} } \\ {1 - \frac{{\lambda_{{\text{t}}} \varepsilon_{{{\text{t}}0}} }}{\varepsilon }} & {\varepsilon_{{{\text{tu}}}} < \varepsilon \le \varepsilon_{{{\text{t0}}}} } \\ 1 & {\varepsilon \le \varepsilon_{{{\text{tu}}}} } \\ \end{array} } \right.$$

where *λ*_t_ = *f*_tr_/*f*_t_ signifies the residual tensile strength coefficient, where *f*_t_ and *f*_tr_ denote the uniaxial tensile and residual tensile strengths, respectively. Additionally, *ε*_t0_ = *f*_t_/*E*_0_ is the elastic tensile strain threshold. Furthermore, *ε*_tu_ = *ηε*_t0_ represents the ultimate tensile strain characterizing the critical state when the complete damage happens. *η* is termed the ultimate strain coefficient.

In addition, when one mesh element fails under compressive loading, *w* can be computed using the following equation:

6 $$w = \left\{ {\begin{array}{*{20}c} 0 & {\varepsilon < \varepsilon_{{{\text{c0}}}} } \\ {1 - \frac{{\lambda_{{\text{c}}} \varepsilon_{{{\text{c0}}}} }}{\varepsilon }} & {\varepsilon \ge \varepsilon_{{{\text{c0}}}} } \\ \end{array} } \right.$$

where *λ*_*c*_ = *f*_*cr*_/*f*_*c*_ represents the residual compressive strength coefficient, where *f*_c_ and *f*_cr_ denote the UCS and residual compressive strength, respectively. *ε*_c0_ = *f*_c_/*E*_*0*_ is the threshold compressive strain.

## Results

**Fig. 3:**
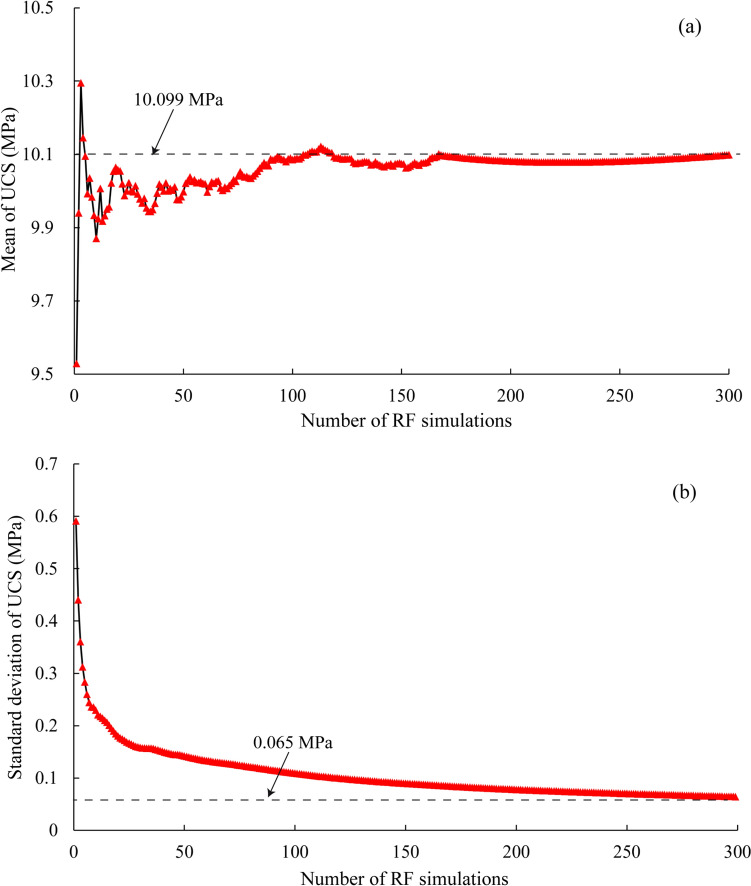
The statistical convergence of UCS during random filed (RF) simulation: **a** mean and **b** standard deviation

To examine the reliability of the repetitive simulation scheme, the statistical convergence of the 300 equivalent random filed simulations is examined, as shown in Fig. 3. It can be seen that intense fluctuation of the mean UCS value exists during the first 50 random field simulations. However, it becomes gradually stable and reaches the final stable value of 10.099 MPa after 300 simulations. Figure 3 also illustrates that the standard deviation of UCS drops dramatically during the first 50 simulations. However, it is gradually stabilized at 0.065 MPa after 280 or more simulations. These results demonstrate that the computation process could successfully converge after 300 equivalent simulations, and the numerical results can be considered statistically reliable.

**Fig. 4:**
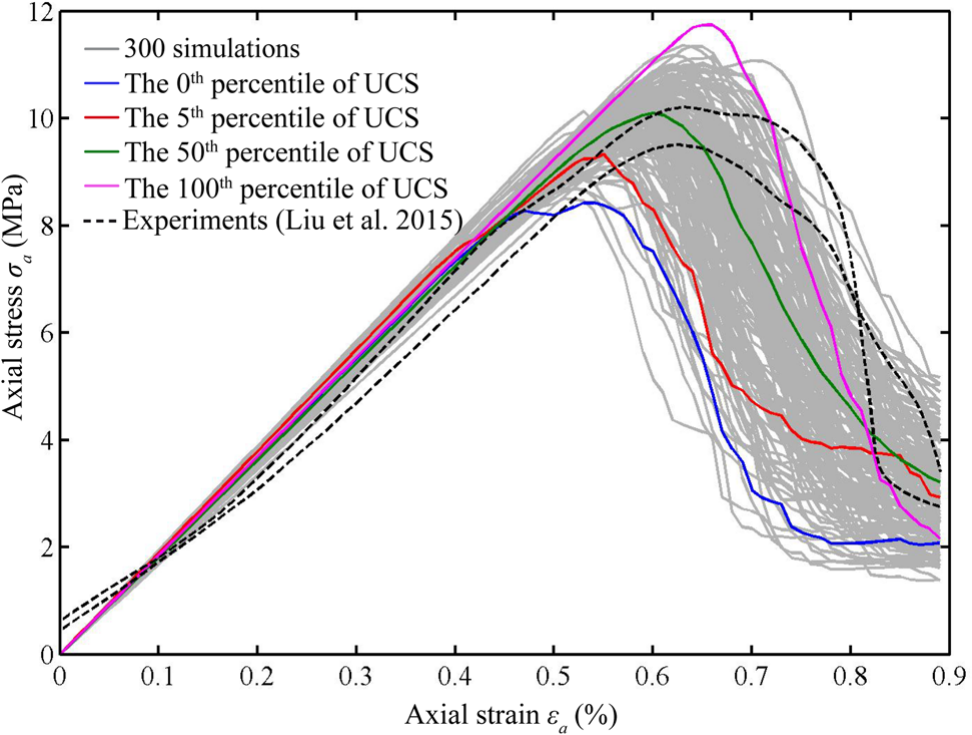
The stress–strain relationships of rock samples under uniaxial compression

Figure 4 depicts the dispersion of stress–strain curves from uniaxial compression tests using the RRFPA method across 300 simulations on rock samples with distinct random fields. For comparison, the experimental data obtained from coal rock samples by Liu et al. (2015) are also included in this figure. To ensure adherence to the quasi-static loading conditions, a small loading rate of 0.1 mm/step was employed in the simulations, as reported by Zhang and Zhao (2014). In Fig. 4, varying colours are utilized to highlight the key percentiles—specifically, the minimum (0^th^ percentile), 5^th^ percentile, median (50^th^ percentile), and maximum (100^th^ percentile) of the UCS across these numerical simulations. The numerical results of stress–strain relationship display a broad distribution that closely aligns with the primary trend observed in the experimental data. Consequently, the overarching mechanical response of the typical coal rock samples used by Liu et al. (2015) can be effectively captured by the series of random RFPA analyses. In fact, the experimental results (Liu et al. 2015) exhibit apparent variability of standard rock testing data on the same type of samples, but varied internal mineral structures. After reaching the peak stresses, the numerical samples demonstrate evident brittle behaviour characterized by rapid stress declines, following the same trend as the experimental data. The random RFPA simulations, incorporating a spectrum of material properties, effectively delineate the upper and lower bounds of stress across all conceivable testing outcomes.

**Fig. 5:**
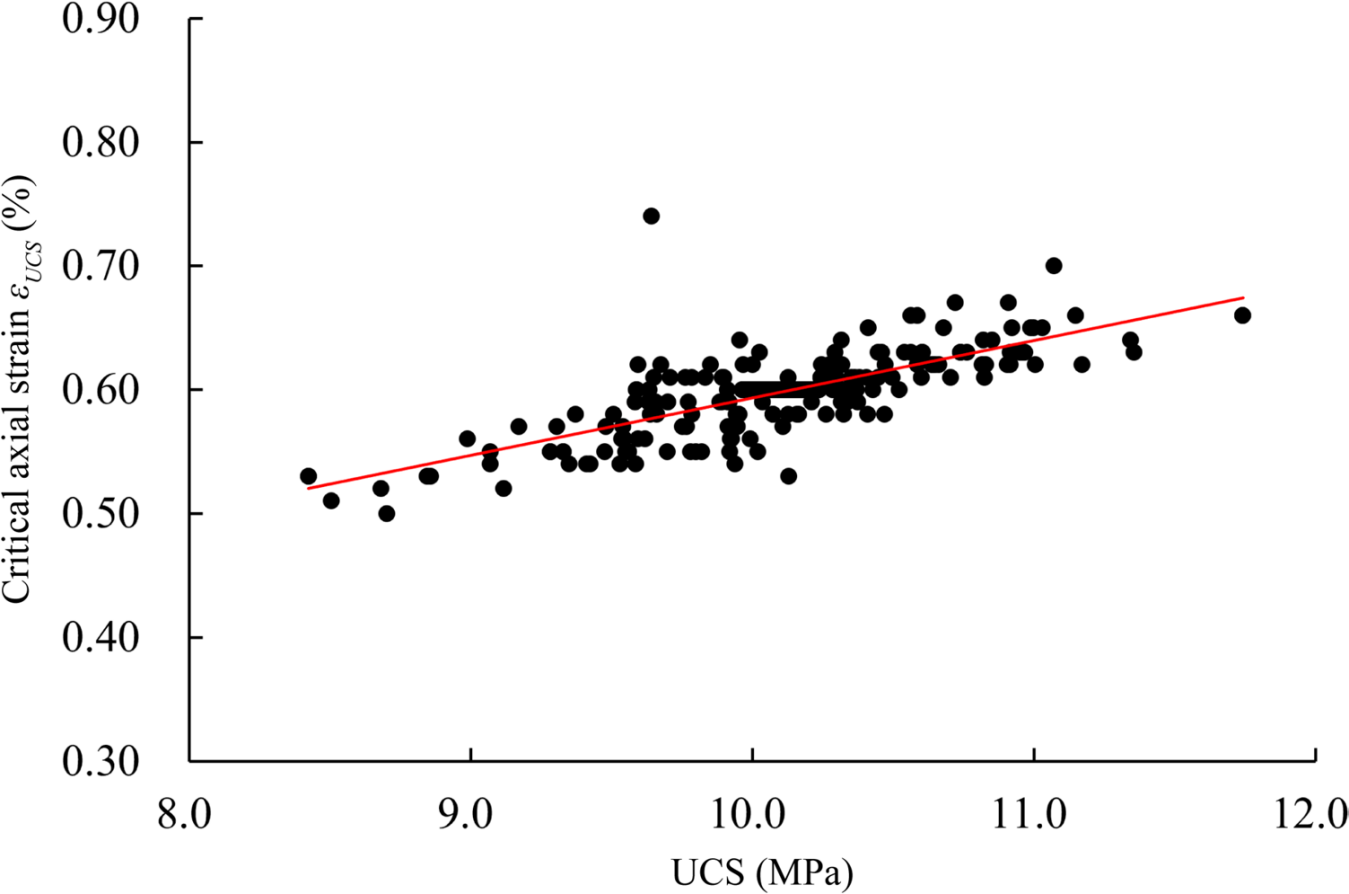
The UCSs and critical axial strains (${\varepsilon }_{\text{UCS}}$) of the 300 simulations

Figure 4 further highlights that, despite variations, the axial strain corresponding to the peak stress point generally increases with the growth of the UCS value. This relationship between the UCS and critical axial strain (${\varepsilon }_{\text{UCS}}$) is depicted in Fig. 5. The overall pattern indicates a positive correlation between UCS and critical axial strain, with the predominant occurrence of ${\varepsilon }_{\text{UCS}}$ falling within the range of 0.55%–0.65%. Notably, the rock sample with UCS value as low as 8.423 MPa can undergo failure at a small critical axial strain ${\varepsilon }_{\text{UCS}}$ = 0.53%, while that with UCS value as high as 11.742 MPa exhibits resistance against considerably larger deformation for ${\varepsilon }_{\text{UCS}}$ = 0.66%.

**Fig. 6:**
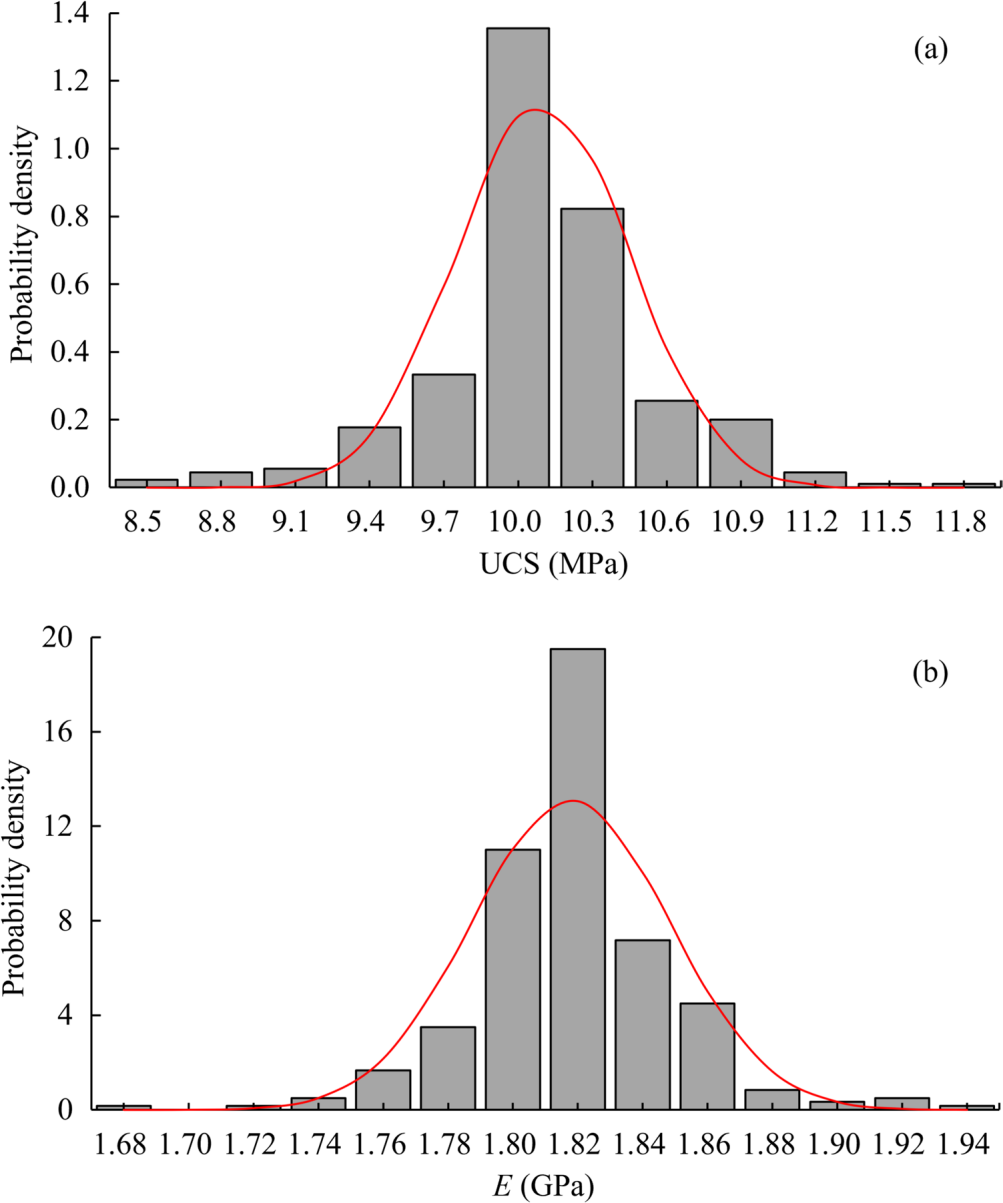
The histograms of **a** UCS and **b** elastic modulus (*E*)

The elastic modulus of rock is determined as the gradient of the initial linear segment of the stress–strain curve corresponding to a strain equals to 0.3%. As depicted in the histograms of UCS and elastic modulus in Fig. 6a, b, across all 300 independent numerical tests, both the UCS and elastic modulus of rock samples follow the normal distributions. The UCS exhibits an average value of 10.099 MPa with a COV of 0.450, while the elastic modulus has an average value of 1.818 GPa with a COV of 0.038. Notably, the average UCS and elastic modulus of the rock samples are smaller than the prescribed statistical mean parameters, indicating that the assumption of uniform material properties used in the traditional finite element methods, can substantially overestimate rock strength and elastic deformation. The differences in COVs between UCS/elastic modulus and their respective random fields (0.3) are attributed to the averaging effect on the variability of material properties.

**Fig. 7:**
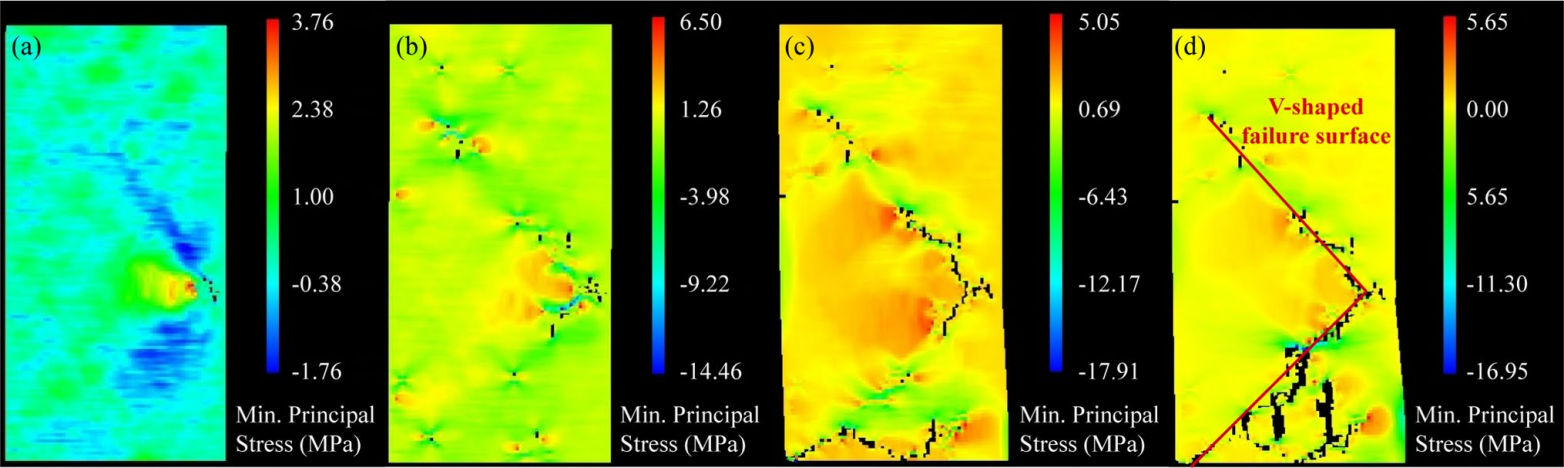
Failure process of the rock sample with the 5^th^ percentile of UCS represented by minimum principal stress contours at different axial strains of **a** *ɛ*_a_ = 0.46%, **b** *ɛ*_a_ = 0.57%, **c** *ɛ*_a_ = 0.74% and **d** *ɛ*_a_ = 0.90%

To illustrate the progressive failure process, the test of 5^th^ percentile of UCS has been selected for detailed analysis as this value normally represents the characteristic material property in real designs. As shown in Fig. 7, the representative V-shaped failure surface can be clearly observed in this model. At a low axial strain of 0.46% (Fig. 7a), a micro-crack formed at the weak zones of the right middle part of the sample. As the axial strain increased to 0.57% (Fig. 7b), more weak elements failed and gradually nucleated to form a localized damage zone at the lower-right-middle part of the sample. Then, in Fig. 7c, the initiated cracks in the damage zone propagated towards the upper and lower left corners at larger axial deformations when the rock sample was close to fail. When the final macro failure occurred, a V-shaped failure surface consisting of micro-cracks was clearly observed within the sample, and the lower block of the sample was fractured into several small rock fragments, as shown in Fig. 7. Besides, Fig. 7 shows that the significant stress concentrations occurred at the cracking tips, leading to the progressive development of cracks. Actually, it was the high concentrated stress that caused the initiation of micro-cracks at the microdefects inside the rock sample. With the formation of initial micro-cracks, the previous concentrated stress was released and transferred to the cracking tips or other microdefects, resulting in further propagation or initiation of more cracks. Namely, it was a continuous process of stress build-up, stress release and stress transfer that caused the creation and development of cracks and the progressive failure of rock sample.

**Fig. 8:**
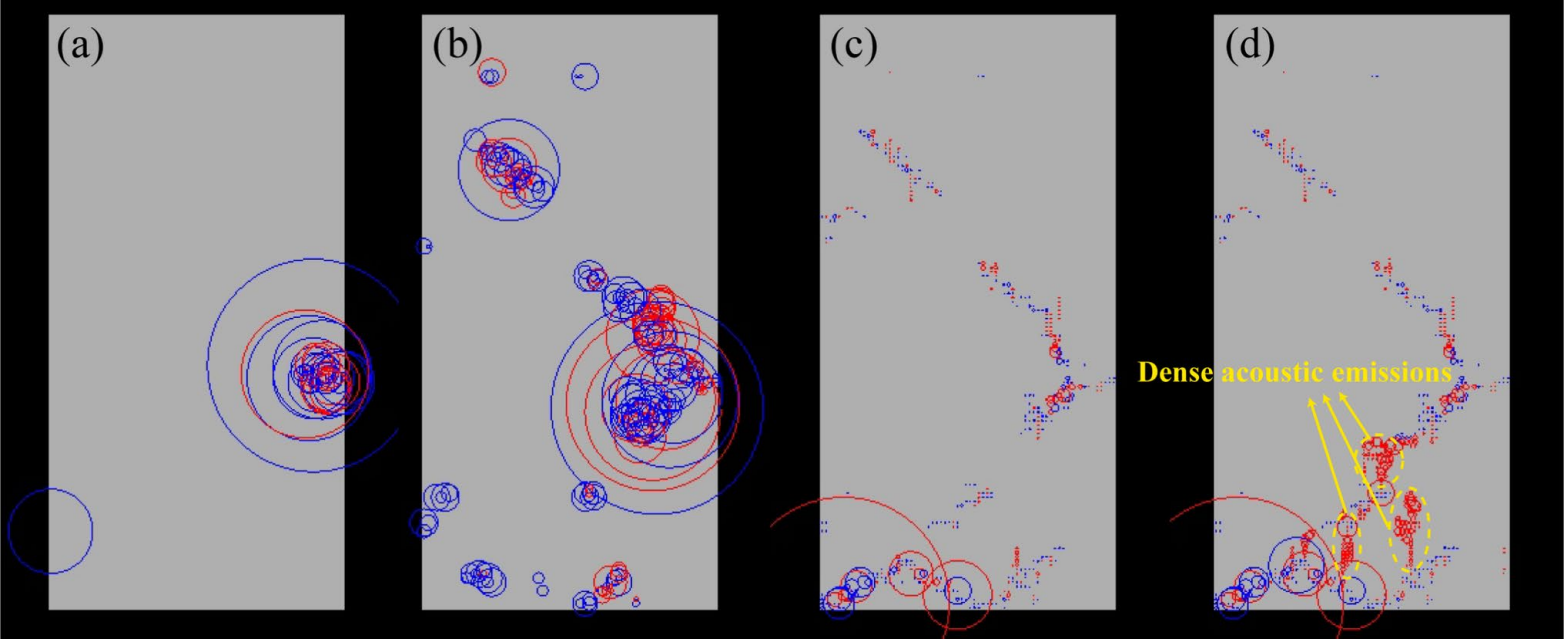
AE release process of the rock sample with the 5^th^ percentile of UCS when the axial strain: **a** *ɛ*_a_ = 0.46%, **b** *ɛ*_a_ = 0.57%, **c** *ɛ*_a_ = 0.74% and **d** *ɛ*_a_ = 0.90% (note: the centre of a circle indicates the location of an AE; the radius of a circle reflects relative energy magnitude; the red and blue colours represent tensile and compressive failures, respectively)

Figure 8 illustrates the spatial distribution of acoustic emissions (AEs) generated during the rock failure process, from which the positional relationship between two individual AE events can be identified, and their relative energy magnitudes can be compared visually. In the RRFPA method, the released energy and position of AEs can be recorded at the moment when the stress/strain state of the elements satisfies the Mohr–Coulomb criterion with a tensile cut-off. In Fig. 8, the centre of a circle indicates the location of an AE, and its radius reflects the relative energy magnitude. The larger radius represents the greater released energy. Meanwhile, the red and blue colours represent the tensile and compressive failures, respectively. Namely, AE events can reflect element damage to a certain level. Figure 8a shows that when the axial strain was 0.46%, many AE events were caused by the formation of the micro-cracks at the right middle part of the sample. Figure 8b further explains the failure modes influenced by the scattered element damages and reveals that high energy was released at the upper left part of the sample due to the occurrence of localized damage. Simultaneously, several shearing failures with small amount of energy occurred at the lower left part of the sample. Figure 8c illustrates that a series of AE events were caused by gradual crack propagations. Especially, the low energy induced by tensile failure at crack tips can be observed. Figure 8d demonstrates that there were three main regions containing dense AEs near the rock bottom, connecting the pre-developed cracks and leading to the final macro failure of the sample.

**Fig. 9:**
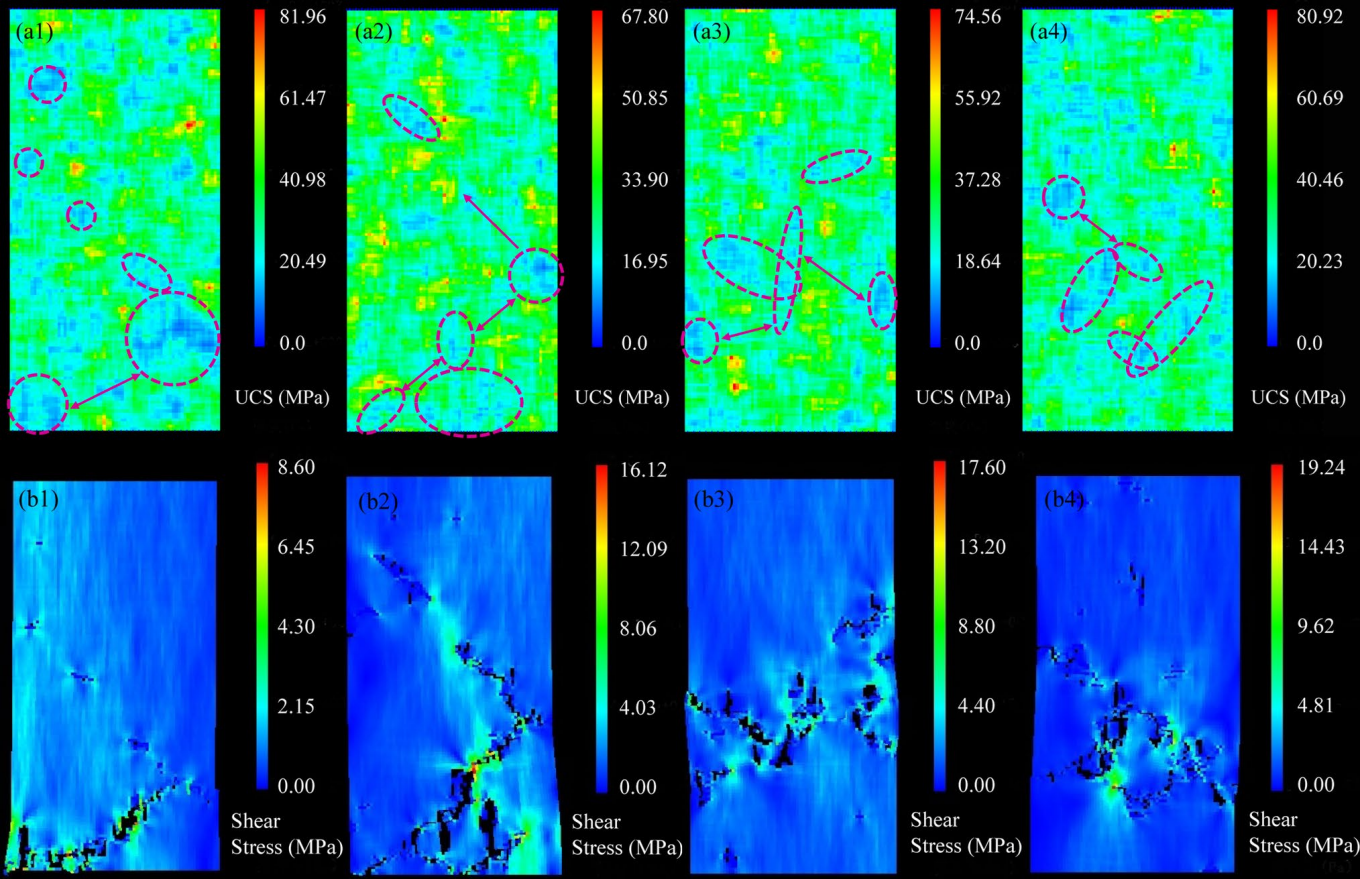
Series **a** the random fields of the initial rock samples and series **b** the final damage zones within the rock samples with different percentiles of UCS including (1) the 0^th^ percentile, (2) the 5^th^ percentile, (3) the 50^th^ percentile and (4) the 100^th^ percentile (note: the dashed red circles in (**a1**)–(**a4**) indicate the main damage zones contributing to final failure)

Figure 9 illustrate the initial random fields of the rock samples with different percentiles of UCS and the corresponding final failure patterns. As the UCS increased, the internal damage zone became gradually flat and distributed closer to the middle region of the sample. For the rock sample shown in Fig. 9a1, b1, although some small and disconnected weak zones existed and resulted in the generation of micro-cracks, it was the two large weak regions located near the bottom that controlled the final failure mode and led to the local instability of the rock sample. Figure 9b2 indicates that with the increase of UCS to the 5^th^ percentile value, more cracks initiated and resulted in the macroscopic rock failure. However, the well-developed cracks at the lower part caused the enlargement of localized damage zone, governing the rock strength. Figure 9a3 illustrates that the scattered weak areas concentrated at the middle part of the sample as the UCS rose. Therefore, many large cracks formed at the middle and propagated gradually to connect with each other, leading to the overall instability of the sample as shown in Fig. 9b3. Figure 9a4 demonstrates that for the highest UCS, only several narrow weak areas existed at the middle region of the sample. Note that the strong area surrounded by weak elements prevented the local failure and improved the rock strength because the generated cracks needed to propagate longer paths to create the final failure surfaces, which consumed more energy. Meanwhile, Fig. 9b4 shows that the high stresses concentrated at the tips of cracks leading to the progressive development of cracks. From Fig. 9b1–b4, it can be seen when the local failure mode of rock sample changed to the overall failure mode, the corresponding macroscopic sample UCS increased gradually.

**Fig. 10:**
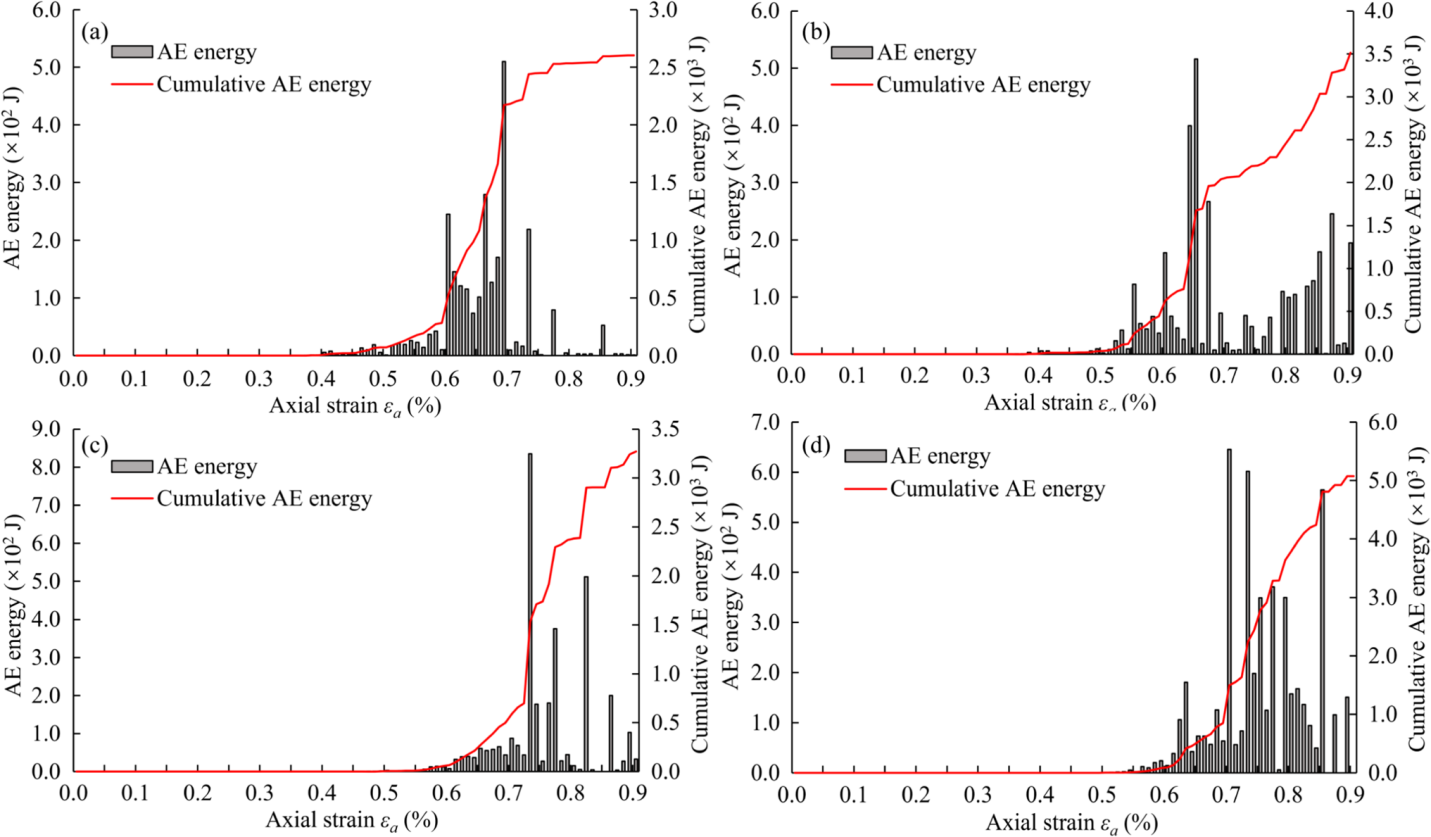
AE energy evolution characteristics of the rock samples with different percentiles of UCS during the compression test: **a** the 0^th^ percentile, **b** the 5^th^ percentile, **c** the 50^th^ percentile, and **d** the 100^th^ percentile

Figure 10 shows the energy evolution characteristics caused by the AE activity of the rock samples with different percentiles of UCS during the compression test. The energy evolution data demonstrates that the concentrated rock damage started to occur when the axial strain exceeded 0.3%. Figure 10a displays a rapid AE release, which was caused by the damaged elements at the bottom of the rock sample with low UCS. The AE energy released by one damaged element can be calculated according to its stress states before and after the occurrence of damage. Then, the cumulative AE energy can be calculated by summing up the AE energy released by all damaged elements. The total cumulative AE energy was only 2.6 × 10^3^ J for the rock sample with the 0^th^ percentile of UCS because of the local instability mode. Figure 10b illustrates that the quick release of AE energy occurred when the axial strain increased from 0.50% to 0.65%. This phenomenon was caused by the formation of the two main cracks inside the rock sample with higher UCS. Simultaneously, it was the serious damage near the bottom of the rock sample that resulted in the gradual AE energy release and final localized instability. Figure 10c indicates that for the rock sample with the 50^th^ percentile of UCS, the maximum AE energy release of 8.5 × 10^2^ J happened when the axial strain was 0.74%. Then, as the axial strain increased, more and more damages appeared at the middle part of the sample and connected to form a large damaged zone, leading to the final rock failure. Figure 10d demonstrates that the damages at the middle part of the rock sample with the 100^th^ percentile of UCS produced relatively uniform and large AE events, and the cumulative AE energy reached a higher value of 5.05 × 10^3^ J. From Fig. 10, it can be summarized that the localized failure pattern would produce faster AE energy release and smaller cumulative AE energy than the overall failure pattern.

**Fig. 11:**
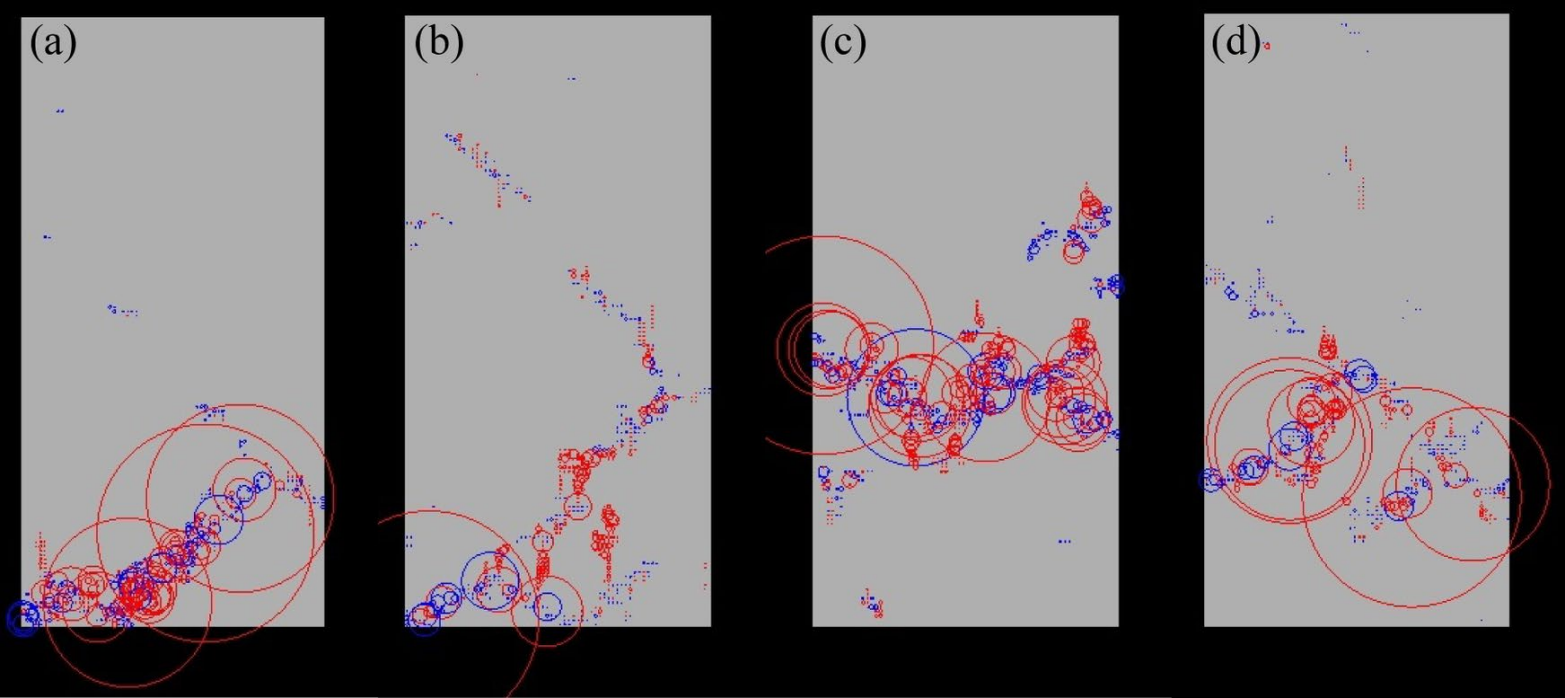
Spatial AE distributions of the rock samples with different percentiles of UCS at the final failure state for rock samples of : **a** the 0^th^ percentile, **b** the 5^th^ percentile, **c** the 50^th^ percentile, and **d** the 100^th^ percentile

Figure 11 shows the spatial AE distributions of the rock samples with different percentiles of UCS. Figure 11a displays that for the rock sample with the 0^th^ percentile of UCS, both tensile and shearing damages occurred near the bottom, and several significant tensile damages released the major AE energy. Figure 11b illustrates that there were several prominent zones characterized by a high concentration of AEs caused by tensile fractures at the lower part of the sample because of its specific random filed realization. This intricately linked to the formation of cracks and ultimately culminated in macroscopic failure. From Fig. 11c, it can be seen that although some serious shearing damages appeared, a series of high-energy AE events were induced by the tensile damages which happened along the middle line of the rock sample with the 50^th^ percentile of UCS. Figure 11d shows that a series of relatively uniform AE events corresponded to an obvious shearing surface at the lower left part of the sample with the highest UCS. In this testing scenario, several large tensile damages produced high AE energy, promoting the final macro failure.

To validate the effectiveness of the proposed RRFPA method, the experimental and numerical data are compared as shown in Table 2. In the table, the absolute and relative errors were calculated by treating the experimental data as the accurate values. Table 2 indicates that the calculated UCS of 10.099 MPa by RRFPA was only 0.061 MPa higher than the experimental value of 10.038 MPa by Liu et al. (2015), and the numerical value by the random discrete element analysis (RDEA) method (Zhao and Liu 2020) was 0.142 MPa higher than the test data. Simultaneously, the elastic modulus of 1.818 GPa and critical axial strain of 0.5556% computed by the RRFPA method were closer to the experimental data (1.806 GPa and 0.5558%) than the simulated values by RDFA (Zhao and Liu 2020). Furthermore, in terms of UCS, elastic modulus and critical axial strain, the maximum relative error of the RRFPA method was only 0.66% which was much less than the maximum relative error of 5.87% by the RDEA method (Zhao and Liu 2020), as shown in Table 2.

**Table 2:** Comparison of the numerical and experimental results

Parameters	UCS (*σ*_c_)/MPa	Elastic modulus (*E*)/GPa	Critical axial strain (${\varepsilon }_{\text{UCS}}$)/%	Absolute error			Relative error/%		
				*σ*_c_/MPa	*E*/GPa	${\varepsilon }_{\text{UCS}}$/%	*σ*_c_	*E*	${\varepsilon }_{\text{UCS}}$
Experiment (Liu et al. 2015)	10.038	1.806	0.5558						
RDEA (Zhao and Liu 2020)	10.18	1.73	0.5884	0.142	−0.076	0.0326	1.41	4.21	5.87
RRFPA	10.099	1.818	0.5556	0.061	0.012	-0.0002	0.61	0.66	0.04

## Conclusions

The traditional RFPA method has been proven effective in modelling the initiation, propagation, and coalescence of rock cracks. However, it does not adequately capture the critical feature that the material properties of rock masses exhibit intrinsic variation and correlation. In this study, the traditional RFPA was enhanced by integrating the random field theory, and the improved method was applied to analyse the mechanical response of rocks during a fundamental uniaxial compression test. The main conclusions are summarized as follows:

1. The RRFPA method was introduced by coupling RFPA and RFT to provide an effective approach to characterize spatial material variability, quantify uncertainties, and enhance the reliability of predictions in rock mechanics. In this method, RFT was utilized to capture the variation of rock parameters as a function of relative distance, enabling the RRFPA to fully account for the influence of intrinsic correlations on fracturing behaviours and failure modes.
2. The RRFPA modelling results exhibited distinct upper and lower bounds of stress across all testing scenarios under uniaxial compressions, which aligned well with the experimental stress–strain relationship. Additionally, after reaching peak stress, the rock samples exhibited pronounced brittle behaviour, characterized by a rapid reduction in stress. A positive correlation between uniaxial compressive strength (UCS) and critical axial strain was observed, with the axial strain predominantly falling within the range of 0.55% to 0.65%.
3. The histograms of UCS and elastic modulus illustrated their adherence to normal distributions. Specifically, UCS had a mean of 10.099 MPa with a COV of 0.450, while elastic modulus had an average of 1.818 GPa with a COV of 0.038. Notably, the average UCS and elastic modulus of the rock samples were considerably lower than the prescribed statistical mean values, suggesting that the uniform material properties tended to overestimate both rock strength and elastic deformation. The localized failure pattern resulted in more rapid AE energy release and a smaller cumulative AE energy than the overall failure pattern.

## Data Availability

The data underpinning this publication can be accessed from Brunel University of London’s data repository, Brunelfigshare here under a CCBY license: 10.17633/rd.brunel.26317462
